# TLR and TNF-R1 activation of the MKK3/MKK6–p38α axis in macrophages is mediated by TPL-2 kinase

**DOI:** 10.1042/BCJ20160502

**Published:** 2016-09-12

**Authors:** Michael J. Pattison, Olivia Mitchell, Helen R. Flynn, Chao-Sheng Chen, Huei-Ting Yang, Hakem Ben-Addi, Stefan Boeing, Ambrosius P. Snijders, Steven C. Ley

**Affiliations:** 1 The Francis Crick Institute, Immune Signalling Laboratory, Mill Hill Laboratory, London NW7 1AA, U.K.; 2 The Francis Crick Institute, Protein Analysis and Proteomics Laboratory, Clare Hall Laboratory, South Mimms EN6 3LD, U.K.; 3 The Francis Crick Institute, Bioinformatics and Biostatistics Service, Lincoln's Inn Fields Laboratory, London WC2A 3LY, U.K.

**Keywords:** IKK, NF-κB1, p38, TLR, TPL-2, tumour necrosis factors

## Abstract

Previous studies suggested that Toll-like receptor (TLR) stimulation of the p38α MAP kinase (MAPK) is mediated by transforming growth factor-β-activated kinase 1 (TAK1) activation of MAPK kinases, MKK3, MKK4 and MKK6. We used quantitative mass spectrometry to monitor tumour progression locus 2 (TPL-2)-dependent protein phosphorylation following TLR4 stimulation with lipopolysaccharide, comparing macrophages from wild-type mice and *Map3k8*^D270A/D270A^ mice expressing catalytically inactive TPL-2 (MAP3K8). In addition to the established TPL-2 substrates MKK1/2, TPL-2 kinase activity was required to phosphorylate the activation loops of MKK3/6, but not of MKK4. MKK3/6 activation required IκB kinase (IKK) phosphorylation of the TPL-2 binding partner nuclear factor κ-light-chain-enhancer of activated B cells (NF-κB1) p105, similar to MKK1/2 activation. Tumour necrosis factor (TNF) stimulation of MKK3/6 phosphorylation was similarly dependent on TPL-2 catalytic activity and IKK phosphorylation of NF-κB1 p105. Owing to redundancy of MKK3/6 with MKK4, *Map3k8*^D270A^ mutation only fractionally decreased lipopolysaccharide activation of p38α. TNF activation of p38α, which is mediated predominantly via MKK3/6, was substantially reduced. TPL-2 catalytic activity was also required for MKK3/6 and p38α activation following macrophage stimulation with *Mycobacterium tuberculosis* and *Listeria monocytogenes*. Our experiments demonstrate that the IKK/NF-κB1 p105/TPL-2 signalling pathway, downstream of TAK1, regulates MKK3/6 and p38α activation in macrophages in inflammation.

## Introduction

Toll-like receptors (TLRs) play a central role in the initiation of innate immune responses to pathogen infection, recognizing invariant pathogen molecules from bacteria, fungi and viruses [1]. For example, lipopolysaccharide (LPS) from Gram-negative bacteria stimulates TLR4 receptors on the plasma membranes of macrophages and dendritic cells, rapidly triggering the expression of multiple genes [2]. The products of these genes can directly target the invading pathogen (e.g. antimicrobial peptides) or induce recruitment of additional immune cells (e.g. cytokines and chemokines). This inflammatory response is essential for direct killing of pathogens by phagocytic cells and the subsequent induction of the adaptive immune response [3].

Induction of gene expression in innate immune cells by all TLRs involves the co-ordinate activation of the IκB kinase (IKK) complex [4], which triggers nuclear translocation of nuclear factor κ-light-chain-enhancer of activated B cell (NF-κB) transcription factors, and each of the major mitogen-activated protein (MAP) kinases (extracellular signal-regulated kinases 1 and 2 [ERK1/2], Jun amino terminal kinases 1 and 2 [JNK1/2] and p38α) [5]. Certain TLRs (e.g. TLR3 and TLR4) also activate interferon regulatory factors, promoting type I interferon (IFN) transcription. In macrophages, TPL-2 (tumour progression locus 2; also known as COT and MAP3K8) is a MAP3 kinase that is essential for activation of ERK1/2 by all TLRs [6]. TPL-2 directly phosphorylates and activates the MAP2 kinases, MKK1 and MKK2, which are upstream of ERK1 and ERK2 MAP kinases (MAPKs) [7]. TPL-2 also mediates ERK1/2 activation by tumour necrosis factor (TNF) receptor 1 (TNF-R1) and interleukin-1 receptor, emphasizing the important role of TPL-2 in innate immune responses [6].

The activation of the ERK1/2 pathway by TPL-2 is directly linked to the activation of NF-κB. TPL-2 forms a stoichiometric complex with NF-κB1 p105, an NF-κB inhibitory protein and the precursor of NF-κB p50 [8]. Binding to NF-κB1 p105 is essential to maintain steady-state levels of TPL-2 and also prevents TPL-2 from interacting with and phosphorylating MKK1 [9,10]. Since all of TPL-2 is associated with p105 in unstimulated macrophages, TLR stimulation must induce the transient release of TPL-2 from p105 to facilitate MKK1/2 and ERK1/2 activation. This is triggered by IKK phosphorylation of p105, which promotes p105 K48-linked ubiquitination and subsequent p105 proteolysis by the proteasome [11,12].

*In vitro* experiments with *Map3k8*^−/−^ macrophages have revealed that TPL-2 signalling has complex effects on cytokine and chemokine expression following TLR4 stimulation, promoting the production of IL-1β, TNF, IL-10, CXCL2, CCL7 and CCL2, while inhibiting IL-12 p70, IFN-β, CXCL10 and CXCL13 [6,13]. Although analyses of cytokine and chemokine regulation by TPL-2 in macrophages indicate that TPL-2 has complex pro-inflammatory and anti-inflammatory effects, disease models in *Map3k8*^−/−^ mice have demonstrated that the net effect of TPL-2 signalling in the innate immune system is to drive pro-inflammatory responses. For example, TPL-2 is required for the development of TNF-induced Crohn's-like inflammatory bowel disease and LPS-induced septic shock [14,15]. TPL-2 is consequently considered a potential drug target in certain autoimmune diseases, particularly those involving TNF [16]. TPL-2 signalling in innate immune cells is also required for effective immune responses to infection with *Listeria monocytogenes*, an intracellular Gram-positive bacterium, and *Mycobacterium tuberculosis* [17]. Negative regulation of IFN by TPL-2 [18] is particularly important for immunity to both of these pathogens.

Comparison of the effects of TPL-2 deficiency with those of small molecule MKK1/2 inhibitors on wild type cells suggests that TPL-2 predominantly regulates the expression of cytokines and chemokines in TLR-stimulated macrophages via activation of the ERK1/2 MAP kinase pathway [6]. However, TPL-2 induces the production of TNF independently of ERK1/2 activation [19], indicating that there are other downstream targets of TPL-2 signalling in macrophages during an innate immune response. To identify novel physiological TPL-2 substrates, we characterized the TPL-2-dependent phosphoproteome in LPS-stimulated primary macrophages by mass spectrometry. This analysis revealed that the catalytic activity of TPL-2 was required for LPS-induced activation loop phosphorylation of the MAP2 kinases, MKK3 and MKK6, in addition to MKK1 and MKK2. Similar to MKK1/2, TPL-2 phosphorylation of MKK3/6, following LPS stimulation, was dependent on IKK phosphorylation of NF-κB1 p105. TNF-induced activation loop phosphorylation of MKK3/6 was also mediated by the IKK/NF-κB1 p105/TPL-2 signalling pathway. Furthermore, TNF activation of p38α, the MAPK phosphorylated by MKK3/6, was dependent on TPL-2 catalytic activity. These results show that a novel output of TPL-2 signalling in the innate immune response of macrophages is mediated by phosphorylation of MKK3 and MKK6, resulting in the activation of the critical pro-inflammatory MAPK p38α.

## Methods

### Mouse strains

Mouse strains were bred in a specific pathogen-free environment at the Francis Crick Institute — Mill Hill Laboratory (London, UK), and all experiments were done in accordance with regulations of the Home Office of the United Kingdom. *Nfkb1*^SSAA/SSAA^ and *Map3k8*^D270A/D270A^ mouse strains have been described previously [20,21] and were all fully backcrossed on to a C57BL/6 background.

### Antibodies and reagents

The 70-mer TPL-2 antibody used for immunoprecipitation of TPL-2 has been described previously [11]. Antibodies against MKK1/2, phospho(S217/S221)-MKK1/2, p38, phospho(T180/Y182)-p38, phospho-T573 RSK (p90 ribosomal S6 kinase), RSK1, phospho-S100 YB-1, YB-1, phospho(Ser133)-CREB (cyclic AMP response element-binding protein), CREB, phospho(Ser376)-MSK1, phospho(S189/S207)-MKK3/6, MKK6, MKK3, MKK4, phospho-MK2 (MAPKAP kinase 2), MK2, phospho(Ser257/Thr261)-MKK4, phospho(Ser271/Thr275)-MKK7, MKK7 JNK, phospho(T184) TAK1 (transforming growth factor-β-activated kinase 1), phospho(S176) IKK, phospho(S935) p105 and p105 were purchased from Cell Signaling Technology. Antibodies to TPL-2 (M20; H-7), Hsp90 and MKK4 were obtained from Santa Cruz. Phospho(T183/Y185)-JNK antibody was purchased from Invitrogen. Recombinant MBP-MKK6^K82A^ and MKK1^D208A^ proteins were obtained through the MRC PPU Reagents and Services facility (MRC PPU, College of Life Sciences, University of Dundee, UK).

### *In vitro* generation and stimulation of macrophages

Bone marrow-derived macrophages (BMDM) were prepared as described previously [11]. For experiments, harvested BMDMs were replated in Nunc tissue culture dishes (six-well plates, 1 × 10^6^ cells/well; 90-mm dishes, 10 × 10^6^ cells) in RPMI 1640 medium (Sigma) supplemented with 1% foetal bovine serum (FBS), antibiotics and 50 µM β-mercaptoethanol. After overnight culture, LPS (*Salmonella enterica* serovar Minnesota R595; Alexis Biochemicals) was added at a final concentration of 100 ng/ml, TNF (Peprotech) at 20 ng/ml, CpG (5′-C-phosphate-G-3′, InvivoGen) at 2 µM and poly(I:C) (polyinosinic-polycytidylic acid, InvivoGen) at 10 µg/ml. Heat-killed *M. tuberculosis* (InvivoGen; 5 µg/ml) and heat-killed *L. monocytogenes* [InvivoGen; 10^7^ heat killed *Listeria monocytogenes* (HKLM)/ml, MOI = 10] were added and plates were centrifuged at 100 ***g*** for 2 min. Control cells were left untreated (time 0). For experiments in which kinase activation was blocked pharmacologically, cells were pre-incubated with 100 nM PD0325901 (MKK1/2) for 10 min, 1 µM VX-745 (p38α) for 1 h or 10 µM BI-605906 (IKK2) for 1 h, prior to stimulation

### SILAC labelling and mass spectrometry

Bone marrow cells were cultured in SILAC BMDM medium [RPMI 1640 medium without arginine and lysine, 10% dialyzed FCS, 20% dialyzed L929 cell conditioned media, light/heavy l-arginine (482 µM), light/heavy l-lysine (799 µM) and methionine (201 µM)] at 8 × 10^6^ cells per 140-mm bacterial petri dish (Sterilin). A low-molecular-weight cut-off was used for FCS (1 kDa; Dundee Cell Products, UK) and L929 conditioned medium (2 kDa MWCO, Slide-A-Lyzer Dialysis flasks, ThermoFisher Scientific). On day 4, non-adherent cells were collected and resuspended in fresh SILAC BMDM medium and added to the plates. On day 6, non-adherent cells were removed and adherent cells were harvested by incubation with 5 ml of phosphate-buffered saline supplemented with 5% FBS and 2.5 mM EDTA. Cells (12 × 10^6^) were seeded per 14 cm tissue culture dish (Nunc) and cultured overnight in SILAC BMDM medium containing 1% dialyzed FCS without L929 supplement.

SILAC-labelled BMDMs were stimulated with 100 ng/ml LPS for 15 min. Cells were lysed in detergent-free lysis buffer [8 M urea, 50 mM Tris–HCl (pH 8.2), 10 mM sodium β-glycerophosphate, 50 mM NaF, 5 mM NaP_2_O_7_, 1 mM EDTA, 1 mM Na_3_VO_4_, 1 mM DTT, 1 mM PMSF, 153 nM aprotinin, 2 µM leupeptin and 100 nM okadaic acid]. Light- and heavy-labelled lysates were mixed 1:1, based on protein concentrations determined using the BCA Protein Assay Kit (ThermoFisher Scientific), to generate mixtures containing a total of 5 mg protein.

SILAC mixtures were reduced and alkylated prior to digestion with trypsin and rLys-C (Promega). Peptide mixtures were desalted by passage through a C_18_ Sep Pak column (Waters) and then fractionated by strong cation exchange using a PolySULFOETHYL A column (PolyLC; 200 Å). Digests were solubilized in buffer A (10 mM ammonium formate, 25% ACN, pH 3.0) and gradient eluted to 80% buffer B (500 mM ammonium formate, 25% ACN, pH 6.8). Phosphopeptides were enriched from each fraction using titanium dioxide beads (Titansphere, GL Sciences), binding in 1 M glycolic acid in 80% (v/v) acetonitrile, 5% (v/v) TFA and eluting with increasing concentrations (1–5%, v/v) of ammonium hydroxide. Dried phosphopeptides were desalted by passage through C_18_ Stage Tips (Thermo Scientific) prior to LC–MS/MS analysis.

Each phosphopeptide-enriched fraction was injected in triplicate for LC–MS/MS analysis. Peptide mixtures were separated using an EASY-Spray column (PepMap RSLC C18, 2 µm) and eluted directly into the LTQ-Orbitrap Velos (ThermoScientific) mass spectrometer. The instrument was operated in data-dependent acquisition mode, with the top 10 most abundant peptides selected for MS/MS fragmentation. The acquired raw mass spectrometric data were processed in MaxQuant [22] (version 1.3.0.5) and peptides/proteins identified using the Andromeda search engine and the *Mus musculus* canonical sequences from UniProtKB. Estimated false discovery rate was set to 1% at the peptide, protein and site level. The MaxQuant output file PhosphoSTY Sites.txt was then imported into Perseus (version 1.4.0.2).

### Immunoprecipitation and immunoblotting

Cells were lysed in HEPES lysis buffer [50 mM HEPES, 100 mM sodium chloride, 50 mM sodium fluoride, 50 mM sodium β-glycerophosphate, 2 mM EDTA, 2 mM EGTA, 10% glycerol, 1 mM sodium orthovanadate, 100 nM okadaic acid, protease inhibitors (Roche Molecular Biochemicals) and 1% Nonidet P-40]. One milligram of cleared lysate was added to 10 µl of Protein A Sepharose fast flow beads (GE Healthcare) covalently coupled with antibodies against MKK3, MKK4 or MKK6, and incubated for 3 h at 4°C. Beads were washed three times with HEPES lysis buffer followed by elution using 10 µl of non-reducing Laemmli buffer and immunoblotting. Immunoblotting of cell lysates was carried out as described previously [11].

### Kinase assays

TPL-2 MKK kinase activity was assayed using a modification of a previously published method [23]. A total of 10 × 10^6^ cells per point were stimulated for 15 min with LPS (100 ng/ml) or left unstimulated, and proteins were extracted using kinase assay lysis buffer [50 mM Tris–HCl (pH 7.5), 1 mM EDTA, 5 mM NaP_2_O_7_, 50 mM NaF, 1% Triton X-100, 100 µM Na_3_VO_4_, 10 mM sodium β-glycerophosphate, 150 mM NaCl, 100 nM okadaic acid, 0.1% 2-mercaptoethanol and 1× protease inhibitor tablet]. Immunoprecipitation from cleared lysates was carried out using a 1:1 mixture of 70-mer (10 µl) and M20 (2 µl; Santa Cruz) TPL-2 antisera. Immunoprecipitates were washed four times in kinase assay lysis buffer, followed by two washes in kinase buffer (50 mM Tris, pH 7.5, 5 mM sodium β-glycerophosphate, 100 nM okadaic acid, 1 mM TCEP, 10 mM MnCl_2_, 0.03% Brij 35). Beads were then resuspended in 50 µl of kinase buffer plus 1 mM ATP and 4 µg of MBP-MKK6^K82A^. Reaction mixtures were incubated at 30°C for 60 min, supernatant removed and immunoblotted using phospho-MKK6 antibody. TPL-2 bound to antibody-coupled beads was immunoblotted, as described previously [11].

Phosphorylation of MKK1 and MKK6 by recombinant TPL-2 protein was determined using 50 nM StrepII-TPL-2^30-404^ [24,25] with 400 nM His6-MKK1^D208A^ and MBP-MKK6^K82A^ substrates, respectively. Assays were performed in kinase buffer [50 mM HEPES (pH 7.4), 1 mM TCEP, 0.01% BSA, 0.01% Brij35, 5 mM MnCl_2_ and 10% DMSO], supplemented with 300 µM ATP ± 10 µM C34 TPL-2 inhibitor [26] for 30 min at 30°C. Substrate phosphorylation was monitored by immunoblotting with phospho-MKK1 or phospho-MKK3/6 antibodies (Cell Signalling).

### Statistical analysis

All data analyses were performed using GraphPad software (GraphPad Software, Inc., San Diego, CA). Data were compared using a two-way ANOVA. *P*-values of <0.05 or 0.005 were considered significant. Error bars represent standard errors of the mean (SEM).

## Results

### Identification of TPL-2-regulated phosphoproteins by mass spectrometry

A mass spectrometry-based quantitative phosphoproteomic analysis was used to identify proteins in LPS-stimulated primary macrophages whose phosphorylation was dependent on TPL-2 catalytic activity [27]. BMDMs were SILAC (stable isotope labelling with amino acids in cell culture) labelled with light arginine/lysine (R0K0) or heavy arginine/lysine (R10K8) amino acids. Using this protocol, incorporation of SILAC amino acids was ≥90% for each BMDM culture, with low arginine/proline conversion. Immunoblotting showed equivalent LPS induction of IKK2, NF-κB1 p105 and MAPK phosphorylation between SILAC-labelled and -unlabelled BMDM (data not shown).

Two pairwise comparisons were made between LPS-stimulated wild-type (WT) BMDMs and *Map3k8*^D270A/D270A^ BMDM, which express catalytically inactive TPL-2 [21]: a forward comparison (mix 2), in which WT cells were labelled with R0K0 (light) medium and *Map3k8*^D270A/D270A^ BMDM with R10K8 (heavy) amino acids, and a reverse comparison (mix 3), in which each cell genotype was labelled with the alternate medium. To control for biological variation for each comparison, lysates were prepared from six independent BMDM cultures from six mice for each genotype and then pooled. A control pairwise comparison between lysates of light-labelled and heavy-labelled LPS-stimulated WT BMDM (mix 1) produced a narrow distribution of phosphosites (Supplementary Figure S1), with only a small fraction showing variation from the mean ratio (mean ± SD = −0.3434 ± 0.4306). This indicated that labelling with SILAC amino acids did not overtly change the BMDM biology, which facilitated the subsequent identification of TPL-2-dependent phosphorylations.

The forward WT versus *Map3k8*^D270A/D270A^ BMDM comparison identified 13 064 phosphosites on 3390 proteins, whereas the reverse comparison identified 11 825 phosphosites on 3253 proteins. Of these, 10 190 phosphosites, corresponding to 3078 proteins, were detected in both mixes. Similar to previous phosphoproteome analyses, 2% of phosphosites were phosphorylated on Tyr, 21% on Thr and 77% on Ser. The full list of all unique phosphosites identified in both forward and reverse comparisons can be found in Supplementary Table S1. In forward and reverse mixes, *Map3k8*^D270A^ mutation reduced the abundance of 116 phosphosites 4-fold or more, while the abundance of 28 phosphosites was increased 4-fold or more. In the following sections, we used the mass spectrometry data to investigate in more detail how TPL-2 signalling regulated the activation of canonical MAPK pathways in TLR-stimulated macrophages.

### *Map3k8*^D270A^ mutation inhibits LPS activation of the ERK1/2 MAPK pathway and its downstream target p90 RSK

**Figure 1.**
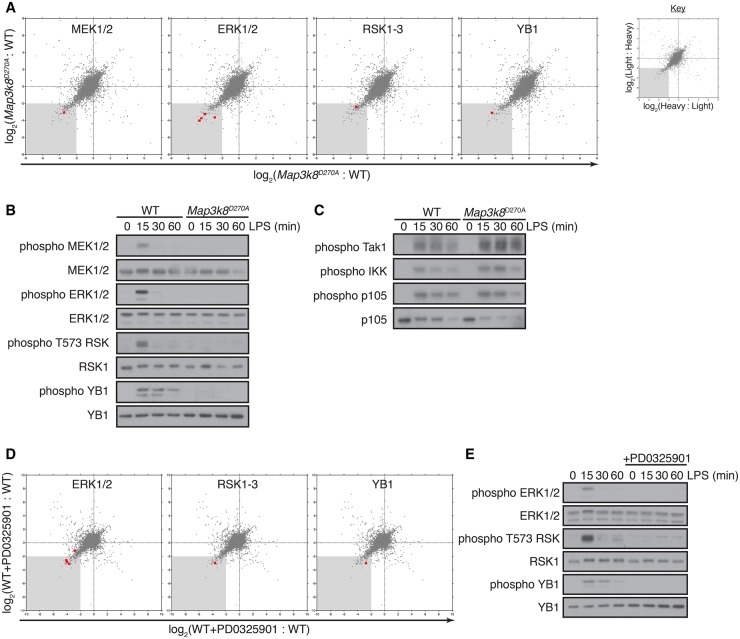
*Tpl2*^D270A^ mutation inhibits LPS activation of the ERK1/2 and p90RSK. (**A**) WT and *Map3k8*^D270A/D270A^ macrophages, labelled with light and heavy or heavy and light (label reversal) SILAC medium, respectively, were stimulated with LPS for 15 min. Light- and heavy-labelled cell lysates were mixed 1:1 (WT:*Map3k8*^D270A/D270A^) based on protein concentration, and phosphosites were quantified by mass spectrometry. Correlation plots show log_2_ D270A/WT ratios for each phosphosite obtained from the two separate SILAC comparisons. The ratios for the label reversal have been inverted. The grey-shaded area indicates 4-fold down-regulation compared with the control. Phosphosites corresponding to specific components of the ERK1/2 MAPK pathway are indicated with red squares and for clarity are indicated in separate correlation plots. (**B** and **C**) WT and *Map3k8*^D270A/D270A^ macrophages were stimulated with LPS for the indicated times. Total cell lysates were immunoblotted for the indicated antigens. Results are representative of three independent experiments. (**D**) WT and WT + PD0325901 macrophages, labelled with SILAC medium were stimulated with LPS for 15 min and phosphopeptides analyzed by mass spectrometry as in (**A**). Correlation plots show log_2_ WT/WT + PD0325901 ratios for each phosphopeptide obtained from two separate SILAC comparisons, with label reversal ratios inverted. The grey-shaded area indicates 4-fold down-regulation compared with the control. Phosphopeptides corresponding to specific components of the ERK1/2 MAPK pathway are indicated with red squares and for clarity are indicated in separate correlation plots. (**E**) WT macrophages ± PD0325901 were stimulated with LPS for the indicated times. Total cell lysates were immunoblotted for the indicated antigens. Results are representative of three independent experiments.

To validate the SILAC-based mass spectrometry method as an effective tool to study TPL-2 signalling, we checked the effect of *Map3k8*^D270A^ mutation on phosphorylation of the well-established direct TPL-2 target proteins, MKK1 (Map2k1; MEK1) and MKK2 (Map2k2; MEK2). Phosphorylation of the activation loops of MKK1 and MKK2, which are identical, was significantly reduced (9-fold) in *Map3k8*^D270A/D270A^ BMDM compared with WT cells (Figure 1A and Supplementary Table S2). In line with this, *Map3k8*^D270A^ mutation also reduced activation loop phosphorylation of the MKK1/2 substrates ERK1 (Mapk3) and ERK2 (Mapk1) 9–20-fold. Mass spectrometric analysis of the TPL-2-dependent phosphoproteome, therefore, was able to confirm that TPL-2 catalytic activity was required for LPS activation of the MKK1/2–ERK1/2 MAPK pathway in macrophages, validating this approach to identify other downstream targets of TPL-2 signalling.

A major output of ERK1/2 signalling in epithelial cells following receptor tyrosine kinase stimulation is mediated by phosphorylation and activation of the MAPK-activated protein kinases (MAPKAP kinases) [28]. These include RSKs and MNKs (MAPK-interacting kinases). Examination of phosphoproteome dataset (Figure 1A and Supplementary Table S2) revealed that *Map3k8*^D270A^ mutation led to a 7-fold reduction in activation loop phosphorylation of the C-terminal kinase domain of RSK on Thr562 (phosphosite identical in RSK1–3), which is known to be a direct target of ERK1/2 required for RSK activation [29]. Consistent with this conclusion, mass spectrometric analyses revealed that *Map3k8*^D270A^ mutation significantly reduced phosphorylation (13-fold) of the transcription factor YB-1 (Y-box 1) on Ser100 (Figure 1A), a site phosphorylated by RSK [30]. No phosphosites corresponding to the activation loop of MNK were identified.

Immunoblotting confirmed that *Map3k8*^D270A^ mutation blocked LPS-induced phosphorylation of MKK1 and ERK1/2 on their activation loops (Figure 1B), as expected [14]. Also, in line with the mass spectrometric data, LPS induction of RSK1 Thr562 was dependent on TPL-2 catalytic activity (Figure 1B), confirming that TPL-2 regulates RSK1 activation in LPS-stimulated macrophages, as previously reported [31]. Immunoblotting also confirmed that phosphorylation of Ser100 YB-1 was substantially reduced in *Map3k8*^D270A/D270A^ BMDM compared with WT cells (Figure 1B). Activation loop phosphorylations of IKK2 were similar between LPS-stimulated *Map3k8*^D270A/D270A^ and WT BMDM (Figure 1C), as was IKK2 phosphorylation of NF-κB1 p105 Ser935 [32]. *Map3k8*^D270A^ mutation slightly elevated activation loop phosphorylation of TAK1 (Figure 1C). Therefore, *Map3k8*^D270A^ mutation did not inhibit phosphorylation of known upstream activators of TPL-2 signalling.

### ERK1/2 activation is the major output of TPL-2 signalling in LPS-stimulated macrophages

We next used mass spectrometry to determine which phosphosites reduced in *Map3k8*^D270A/D270A^ macrophages compared with WT were changed due to altered ERK1/2 activation. Two pairwise comparisons (mixes 4 and 5) were made between LPS-stimulated WT BMDMs cultured in the absence or presence of the specific MKK1/2 inhibitor PD0325901 [33]. The full list of all unique phosphosites identified in both forward and reverse comparisons can be found in Supplementary Table S1.

As expected, PD0325901 reduced phosphorylation of ERK1 (Thr203 and Tyr205) and ERK2 (Thr183 and Tyr185) on their activation loops in LPS-stimulated BMDM. Phosphorylation of Thr562 RSK1 and Ser100 YB-1 was also inhibited by PD0325901 treatment (Figure 1D and Supplementary Table S2). Immunoblotting of BMDM cell lysates confirmed that LPS induction of each of these phosphorylations was substantially reduced by PD0325901 treatment (Figure 1E). These results suggested that *Map3k8*^D270A^ mutation reduced activation of RSK1 and phosphorylation of its downstream substrate YB-1 by preventing ERK1/2 activation, consistent with earlier experiments with serum-stimulated fibroblasts [29].

**Figure 2.**
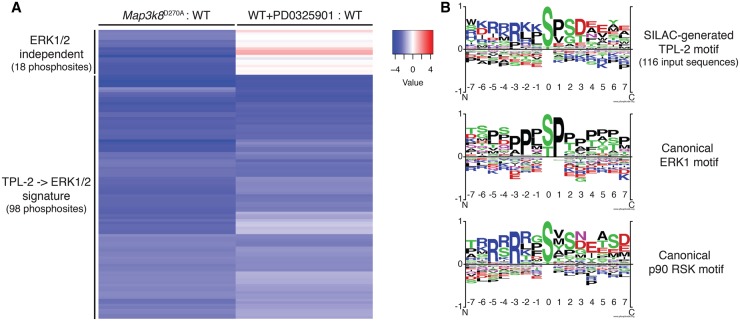
ERK1/2 activation is the major output of TPL-2 signalling following LPS stimulation of macrophages. (**A**) Heatmap showing log_2_ ratios averaged from both WT/*Map3k8*^D270A/D270A^ and WT ± PD0325901 comparisons (Figure 1) for all phosphopeptides ≥4× down-regulated by *Map3k8*^D270A^ mutation. Data underwent hierarchical clustering by rows and column. (**B**) TPL-2 phosphorylation motif deduced from analysis of all phosphopeptides ≥4× down-regulated by *Map3k8*^D270A^ mutation. Canonical ERK1 and RSK phosphorylation motifs from published studies are also shown for comparison.

TPL-2-induced phosphosites, identified in the *Map3k8*^D270A^/WT experiment, were next compared with ERK1/2-dependent phosphosites, identified in the WT ± PD0325901 experiment. *Map3k8*^D270A^ mutation and PD0325901 treatment had largely similar effects, with considerable overlap in down-regulated phosphosites (Figure 2A and Supplementary Table S3). Thus, the major output of TPL-2 signalling in LPS-stimulated macrophages was mediated by the activation of ERK1/2. However, TPL-2 also regulated the phosphorylation of a few proteins (18 phosphosites from 15 proteins) independently of ERK1/2 activation, since the abundance of many phosphosites was decreased ≥4-fold by *Map3k8*^D270A^ mutation but unaffected by PD0325901 treatment (Figure 2A and Supplementary Table S3). This suggested that TPL-2 mediated some of its functions in innate immune responses independently of ERK1/2 activation.

Analysis of the phosphosites reduced ≥4-fold by *Map3k8*^D270A^ mutation revealed a strong preference for proline after the phosphorylated Ser or Thr residue (Figure 2B, top panel), consistent with TPL-2 predominantly regulating phosphorylation directly by ERK1 and ERK2, which are proline-directed kinases (Figure 2B, middle panel) [34]. However, there was also enrichment at the −2 to −4 positions for basic residues (Arg/Lys) and for acidic residues in the +3 to +6 positions (Asp/Glu) in the SILAC-generated TPL-2 motif, similar to the optimal phosphorylation motif for RSK (Figure 2B, lower panel) [35]. This suggested that TPL-2 additionally modulates protein phosphorylation via RSK, downstream of ERK1/2. Our analyses of published phosphoproteomic datasets investigating the effect of MKK1/2 inhibitors in growth factor- or serum-stimulated epithelial cells [36,37] generated similar motifs that were a composite of ERK1/2 and RSK motifs. Noticeably, the motif generated from the phosphosites regulated by TPL-2 independently of ERK1/2 did not resemble that seen for MAPKs, suggesting that TPL-2 does not regulate the phosphorylation of these proteins via MAPK activation (Supplementary Figure S2). In contrast, the motif generated from the 98 phosphosites similarly affected by *Map3k8*^D270A^ mutation and PD0325901 treatment strongly resembled the overall TPL-2 motif, with similarities to both ERK1/2 and RSK phosphorylation motifs.

Functional classification of proteins whose phosphorylation was reduced ≥4-fold by *Map3k8*^D270A^ mutation based on UniProt annotations (Supplementary Figure S3 and Supplementary Table S4) revealed that TPL-2 signalling regulated phosphorylation of proteins in multiple cellular compartments (including the cytoplasm, nucleus and membrane), which were involved in many cellular processes (e.g. RNA processing, transcription, intracellular transport, autophagy and cell cycle regulation).

### TPL-2 regulates MKK3/6 activation in LPS-stimulated macrophages

**Figure 3.**
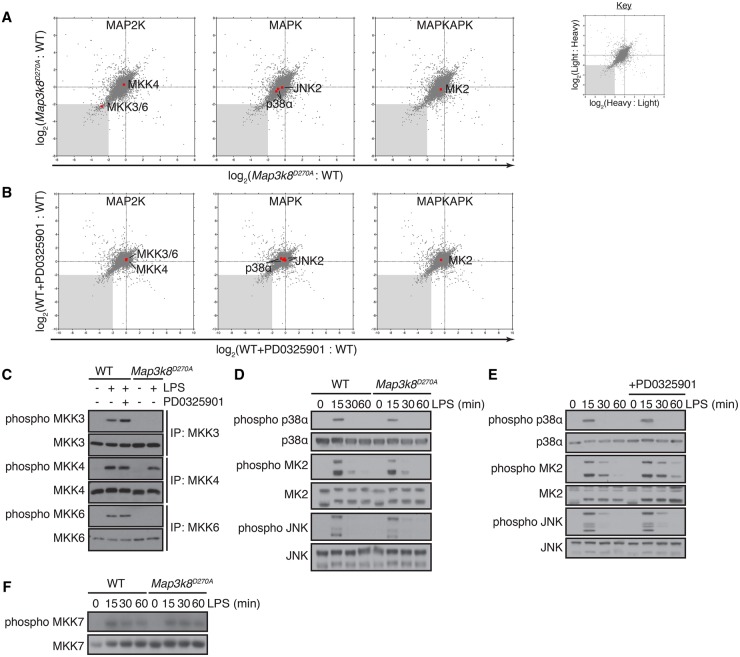
*Map3k8*^D270A^ mutation inhibits activation loop phosphorylation of MKK3 and MKK6 induced by LPS stimulation. (**A** and **B**) Phosphopeptides corresponding to p38α and JNK MAPKs and their upstream MAP2 kinases, MKK3/6 and MKK4, respectively, are indicated on the correlation plots from the WT/*Map3k8*^D270A/D270A^ and WT ± PD0325901 experiments described in Figure 1A,D. The phosphopeptide corresponding to the activation loop of MK2, which is directly phosphorylated by p38α, is also shown. (**C**) MKK3, MKK4 and MKK6 were immunoprecipitated from WT and *Map3k8*^D270A/D270A^ macrophages stimulated with LPS ± PD0325901 for 15 min. Immunoprecipitates were immunoblotted for the indicated antigens. Results are representative of three independent experiments. (**D**) WT and *Map3k8*^D270A/D270A^ macrophages and (**E**) WT macrophages ± PD0325901 were stimulated with LPS for the indicated times. Total cell lysates were immunoblotted for the antigens indicated to the left of the panels. (**F**) WT and *Map3k8*^D270A/D270A^ macrophages were stimulated with LPS for the indicated times. Total cell lysates were immunoblotted for the indicated antigens. Results are representative of three independent experiments.

Proteins whose phosphorylation was reduced by *Map3k8*^D270A^ mutation independently of ERK1/2 activation could include novel physiological targets of TPL-2. Of particular interest, since TPL-2 functions as an MAP3K [7], phosphorylations of the identical activation loops of the MAP2 kinases, MKK3 and MKK6, were reduced 6-fold by *Map3k8*^D270A^ mutation (Figure 3A and Supplementary Table S5), but unaffected by PD0325901 treatment (Figure 3B and Supplementary Table S5). Activation loop phosphorylation of MKK4 was also unaltered by PD0325901 and similar between *Map3k8*^D270A/D270A^ BMDM and WT cells (Figure 3A and Supplementary Table S5). No MKK7 phosphosites were identified. However, immunoblotting of BMDM lysates revealed that the LPS induction of MKK7 activation loop phosphorylation was not affected by *Map3k8*^D270A^ mutation (Figure 3F). These results suggested that TPL-2 catalytic activity could regulate LPS activation of p38α in BMDM, independently of ERK1/2 activation, by controlling the activation of the p38α kinases MKK3/6. Consistent with this hypothesis, mass spectrometric analyses (Figure 3A and Supplementary Table S5) revealed that *Map3k8*^D270A^ mutation reduced activation loop phosphorylations of p38α (35% reduction) and its downstream substrate MK2 (20% reduction) [28]. p38α and MK2 phosphorylation were not affected by PD0325901 treatment (Figure 3B and Supplementary Table S5), in line with the normal phosphorylation of MKK3/6.

To investigate further the importance of TPL-2 catalytic activity for LPS-induced phosphorylation of the MAP2 kinases upstream of p38α, MKK3, MKK4 and MKK6 were separately immunoprecipitated from *Map3k8*^D270A/D270A^ and WT BMDM lysates and then immunoblotted with phospho-MKK antibodies that recognize the activation loops of each MAP2 kinase. Consistent with the mass spectrometry data, *Map3k8*^D270A^ mutation blocked LPS-induced phosphorylation of MKK3 and MKK6, while MKK4 phosphorylation was not affected (Figure 3C). Pretreatment of WT BMDM with the MKK1/2 inhibitor PD0325901 showed that LPS-induced phosphorylation of MKK3, MKK4 and MKK6 was independent of ERK1/2 activation. Immunoblotting of BMDM lysates confirmed that LPS-induced phosphorylations of the p38α and MK2 activation loops were fractionally reduced by *Map3k8*^D270A^ mutation (Figure 3D), but were not changed by PD0325901 treatment (Figure 3E). JNK1/2 activation loop phosphorylation was not altered by either *Map3k8*^D270A^ mutation or PD0325901 (Figure 3D,E). Together, these results demonstrated that *Map3k8*^D270A^ mutation blocked LPS induction of MKK3/6 phosphorylation separately from any inhibitory effects on ERK1/2 MAPK pathway activation, resulting in a partial reduction in p38α activation.

**Figure 4.**
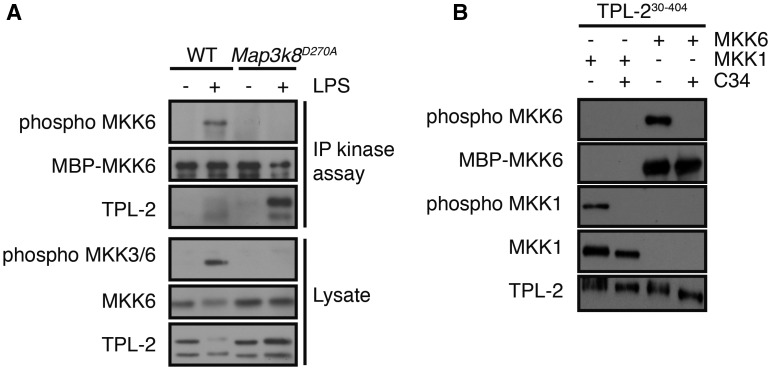
TPL-2 directly phosphorylates the activation loop of MKK6. (**A**) TPL2 was immunoprecipitated from lysates of WT and *Map3k8*^D270A/D270A^ macrophages ± LPS stimulation and assayed for its ability to phosphorylate MBP-MKK6^K82A^ *in vitro*. Activation loop phosphorylation of MBP-MKK6^K82A^ was monitored by immunoblotting with phospho-MKK6 antibody. Immunoblotting of eluates from anti-TPL-2 beads shows relative amounts of TPL-2 in each immunoprecipitate. Endogenous MKK6 phosphorylation was monitored by immunoblotting of cell lysates. (**B**) Recombinant TPL-2^30-404^ was incubated with His6-MKK1^D208A^ or MBP-MKK6^K82A^ substrates plus ATP ± C34 TPL-2 inhibitor. Activation loop phosphorylation and loading of His6-MKK1^D208A^ and MBP-MKK6^K82A^ were assessed by immunoblotting. Results are representative of at least three similar experiments.

One explanation for the dependence of MKK3/6 phosphorylation on TPL-2 catalytic activity was that TPL-2 functioned as the essential MKK3/6 kinase in LPS-stimulated macrophages. Consistent with this hypothesis, TPL-2 immunoprecipitated from WT BMDM phosphorylated recombinant MKK6 on its activation loop *in vitro* after LPS stimulation (Figure 4A). In contrast, no MKK6 kinase activity was associated with kinase inactive TPL2^D270A^ immunoprecipitated from LPS-stimulated *Map3k8*^D270A/D270A^ macrophages, confirming that TPL-2 itself, and not a co-purifying kinase, was responsible for the phosphorylation of MKK6. Purified recombinant TPL-2^30-404^ was also able to phosphorylate the activation loop of recombinant MKK6 protein *in vitro* and this was blocked by the addition of the specific TPL-2 inhibitor C34 [26] to the kinase assay (Figure 4B). Together, these results indicated that TPL-2 functioned as a direct MKK3/6 kinase in LPS-stimulated macrophages, in addition to its established role as a MKK1/2 kinase.

### LPS activation of MKK3 and MKK6 in macrophages is dependent on IKK phosphorylation of NF-κB1 p105

In previous work, we established that LPS activation of TPL-2 MKK1 kinase activity in macrophages requires its release from NF-κB1 p105, triggered by IKK-induced p105 proteolysis [11]. We next investigated whether LPS activation of MKK3/6 kinase activity was regulated in a similar fashion.

**Figure 5.**
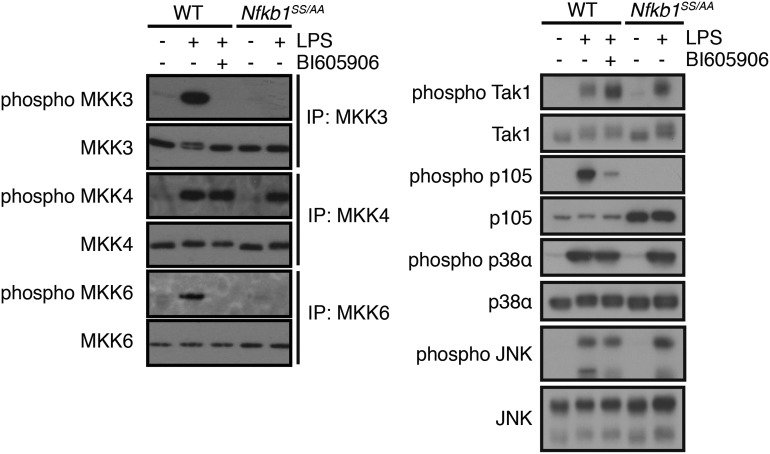
LPS activation of MKK3 and MKK6 is dependent on IKK phosphorylation of NF-κB1 p105. WT and *Nfkb1*^SSAA/SSAA^ macrophages ± BI605906 were stimulated with LPS for 30 min. In the left-hand panel, the MAP2 kinases shown were immunoprecipitated (IP) from cell lysates and immunoblotted for the antigens indicated to the left of the panels. In the right-hand panel, total cell lysates were immunoblotted directly. Results are representative of three independent experiments.

Immunoblotting of MKK immunoprecipitates demonstrated that activation of MKK3 and MKK6 was blocked by pharmacological inhibition of IKK2 with BI605906 [38], whereas MKK4 activation was not (Figure 5, left-hand panels). Immunoblotting of BMDM lysates showed that BI605906 reduced LPS induction of IKK2 phosphorylation of NF-κB1 p105 on Ser935 (Figure 5, right-hand panels), as expected [32]. However, BI605906 did not affect LPS-induced phosphorylation of Thr184 in the activation loop of TAK1, the MAP3 kinase upstream of IKK [39,40]. To determine whether IKK2 phosphorylation of NF-κB1 p105 was required for LPS activation of MKK3 and MKK6, BMDMs were prepared from *Nfkb1*^SSAA^ mice in which the IKK target sites on p105 are mutated to alanine [19,20]. Immunoblotting of cell lysates and immunoprecipitates demonstrated that LPS activation of MKK3/6 was blocked by *Nfkb1*^SSAA^ mutation, while TAK1, MKK4, p38α and JNK activation were minimally affected (Figure 5). LPS activation of MKK3 and MKK6, therefore, was dependent on IKK complex phosphorylation of NF-κB1 p105. This indicated that activation of TPL-2 kinase activity for MKK3/6 was regulated by the IKK/NF-κB1 p105 signalling pathway, similar to MKK1/2 [11,19].

### TPL-2 catalytic activity is required for MKK3/6 and p38α activation by TNF-R1 in macrophages

p38α can be phosphorylated and activated by MKK3, MKK4 and MKK6 *in vitro* [41,42]. Furthermore, blockade of p38α activation by ultraviolet (UV) radiation in fibroblasts requires MKK4 knockdown, as well as the combined deficiency of MKK3 and MKK6 [43]. Thus, UV activation of p38α in cells is mediated by MKK3, MKK4 and MKK6. In LPS-stimulated BMDM, we found that *Map3k8*^D270A^ mutation inhibited activation of MKK3 and MKK6, but not of MKK4, and only fractionally reduced p38α activation (Figure 3A,C). This suggested that, similar to UV activation of p38α in fibroblasts, MKK4 functioned redundantly with MKK3 and MKK6 to activate p38α following TLR4 stimulation of macrophages.

**Figure 6.**
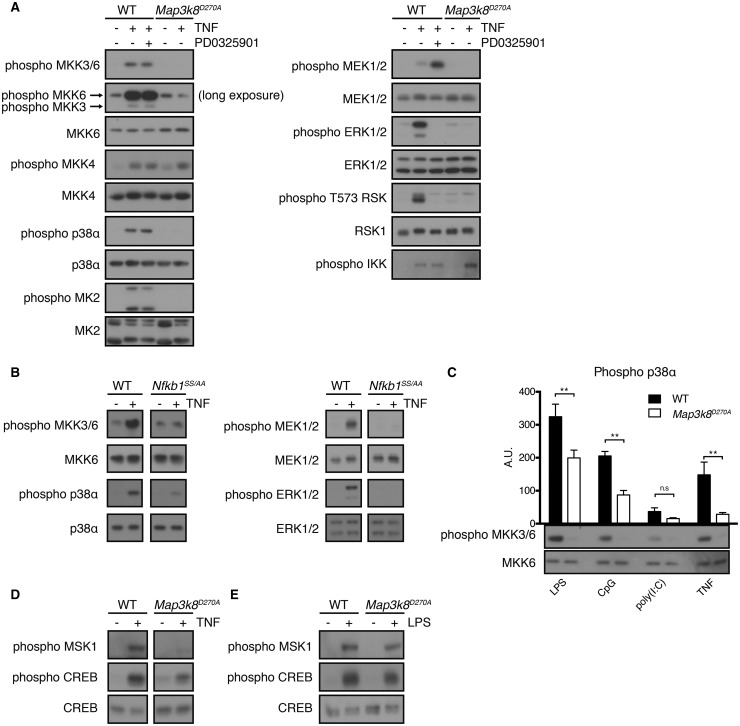
TPL-2 catalytic activity is required for TNF activation of MKK3/6, p38α and MK2 in macrophages. (**A**) WT and *Map3k8*^D270A/D270A^ macrophages stimulated with TNF ± PD0325901 for 15 min. Total cell lysates were immunoblotted for the indicated antigens. (**B**) WT and *Nfkb1*^SSAA/SSAA^ macrophages were stimulated with TNF for 15 min. Total cell lysates were immunoblotted for the indicated antigens. (**C**) WT and *Map3k8*^D270A/D270A^ macrophages were stimulated separately with a panel of agonists. Activation loop phosphorylation of p38α was quantified by infrared scanning of immunoblots. Data, normalized to total p38α levels, are plotted (*n* = 5; SEM). *P*-values were calculated by two-way ANOVA. **P ≤ 0.005. MKK6 phosphorylation was determined by immunoblotting of total cell lysates (lower panels). (**D**) WT and *Map3k8*^D270A/D270A^ macrophages stimulated with TNF for 15 min. Total cell lysates were immunoblotted for the indicated antigens. (**E**) WT and *Map3k8*^D270A/D270A^ macrophages stimulated with LPS for 15 min. Total cell lysates were immunoblotted for the indicated antigens. Immunoblots in panels **A**, **C**, **D** and **E** are representative of at least three independent experiments, and in panel **B** representative of two independent experiments.

In contrast with UV stimulation, phosphorylation and activation of p38α following TNF stimulation of fibroblasts are mediated only by MKK3 and MKK6, with MKK4 unable to substitute for MKK3/6 deficiency [43]. We therefore investigated the effect of *Map3k8*^D270A^ mutation on TNF activation of MAPK pathways in macrophages. Immunoblotting of BMDM lysates revealed that *Map3k8*^D270A^ mutation substantially reduced activation loop phosphorylation of MKK3 and MKK6 stimulated by TNF (Figure 6A, left-hand panel). Significantly, *Map3k8*^D270A^ mutation also blocked TNF-induced activation loop phosphorylation of p38α and MK2. PD0325901 had no inhibitory effect on the phosphorylation of MKK3/6, p38α and MK2 induced by TNF (Figure 6A, left-hand panel), indicating that the inhibitory effects of *Map3k8*^D270A^ mutation on TNF activation of the p38α MAPK pathway were independent of ERK1/2 activation. Neither *Map3k8*^D270A^ mutation nor PD0325901 treatment affected TNF-induced activation loop phosphorylation of IKK2 (Figure 6A, right-hand panel), upstream of TPL-2, or MKK4 (Figure 6A, left-hand panel). ERK1/2 and RSK T573 activation loop phosphorylations induced by TNF were blocked by *Map3k8*^D270A^ mutation and PD0325901 (Figure 6A, right-hand panel), as expected [44]. *Nfkb1*^SSAA^ mutation also reduced TNF activation of MKK3/6 and p38α (Figure 6B, left-hand panels), as well as blocking MKK1/2 and ERK1/2 activation (Figure 6B, right-hand panels), as shown previously [19]. Together, these results demonstrated that TNF activated p38α MAPK via TPL-2-dependent phosphorylation of MKK3/6, using the IKK/NF-κB1 p105/TPL-2 signalling pathway.

The activation of mitogen- and stress-activated protein kinases (MSKs) requires phosphorylation by both ERK1/2 and p38α MAPKs [45]. Since *Map3k8*^D270A^ mutation substantially reduced TNF activation of ERK1/2 and p38α, it was expected that MSK activation would be similarly impaired. Consistent with this, immunoblotting of BMDM lysates demonstrated that phosphorylation of MSK on Ser376 was strongly reduced in *Map3k8*^D270A/D270A^ BMDM, compared with WT, following TNF stimulation. In contrast, the reduction in MSK Ser376 phosphorylation after LPS stimulation was less pronounced, in line with the fractional decrease in p38α activation (Figure 3D). Similarly, phosphorylation of the transcription factor CREB at Ser133 by MSK [46,47] was clearly reduced by *Map3k8*^D270A^ mutation following TNF stimulation of BMDM (Figure 6D), while the reduction following LPS was marginal (Figure 6E).

TPL-2 is required for activation of MKK1/2-ERK1/2 by multiple TLR ligands in macrophages [6]. To investigate whether this was also the case for activation of MKK3/6 and p38α, we stimulated WT and *Map3k8*^D270A/D270A^ BMDM separately with poly(I:C) (TLR3) and CpG (TLR9). Similar to LPS and TNF stimulation, *Map3k8*^D270A^ mutation blocked activation loop phosphorylation on MKK3/6 following stimulation with both of these agonists (Figure 6C, lower panels). CpG induction of p38α activation loop phosphorylation was significantly reduced (by 50–60%) in *Map3k8*^D270A/D270A^ BMDM compared with WT (Figure 6C, graph). Poly(I:C) activation of p38α was consistently reduced by *Map3k8*^D270A^ mutation, but this difference did not reach statistical significance. *Map3k8*^D270A^ mutation significantly reduced by ∼30% p38α activation following LPS stimulation and by ∼80% following TNF stimulation. Thus, TPL-2 is an essential MAP3 kinase for MKK3 and MKK6 downstream of multiple TLRs and TNF-R1 in macrophages, variably contributing to overall p38α activation depending on the stimulated receptor.

### *M. tuberculosis* and *L. monocytogenes* activate MKK3/6 and p38α in macrophages via TPL-2

Our previous work established that the immune responses to *M. tuberculosis* and *L. monocytogenes* are significantly impaired in *Map3k8*^−/−^ mice compared with WT controls [17]. Analyses of *Map3k8*^−/−^ *Rag1*^−/−^ and *Rag1*^−/−^ mice indicated that TPL-2 is required for optimal innate immune responses to both of these Gram-negative bacteria. Macrophages and other phagocytes have been shown to be the major infected cell types for both pathogens [48,49]. The results of the present study suggested that TPL-2 activation of p38α might be involved in the initial innate immune response of macrophages to these pathogens.

**Figure 7.**
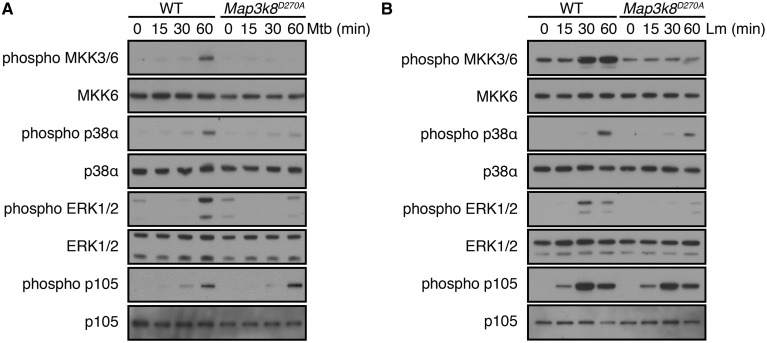
Activation of MKK3/6 and p38α by *M. tuberculosis* and *L. monocytogenes* is reduced by *Map3k8*^D270A^ mutation. (**A** and **B**) WT and *Map3k8*^D270A/D270A^ macrophages were stimulated with heat-killed *M. tuberculosis* (**A**) or *L. monocytogenes* (**B**) for the times shown. Total cell lysates were immunoblotted for the indicated antigens. Results are representative of two independent experiments.

To investigate this possibility, WT and *Map3k8*^D270A/D270A^ BMDM were separately cultured with heat-inactivated *M. tuberculosis* or *L. monocytogenes*. Activation loop phosphorylations of ERK1/2 induced by *M. tuberculosis* or *L. monocytogenes* were reduced by *Map3k8*^D270A^ mutation (Figure 7A,B), as expected from previous studies [17,50]. Both bacteria induced the activation loop phosphorylation of MKK3/6 and p38α in WT cells. However, induction of MKK3/6 phosphorylation was blocked in *Map3k8*^D270A/D270A^ BMDM, and p38α phosphorylation was substantially reduced. *Map3k8*^D270A^ mutation did not impair Ser935 phosphorylation of NF-κB1 p105 by either bacteria, showing that TPL-2 catalytic activity was not required for IKK2 activation. These results indicate that TPL-2 activated p38α and ERK1/2 following the initial interaction of *M. tuberculosis* and *L. monocytogenes* bacteria with macrophages. Given the known importance of p38α and ERK1/2 in controlling cytokine/chemokine gene expression in TLR-stimulated macrophages [5], activation of both MAPKs is probably required for TPL-2 to promote optimal innate immune responses to these pathogens [17].

## Discussion

**Figure 8.**
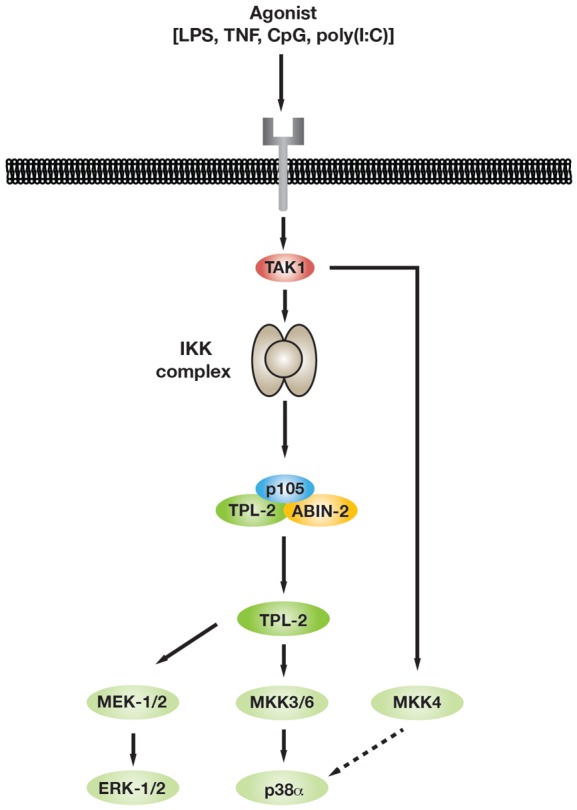
TLR signalling pathways regulating p38α MAPK activation. Schematic diagram of p38α activation pathway activated in innate immune responses.

The present study has demonstrated that, in addition to its established substrates MKK1 and MKK2, a novel major output of TPL-2 signalling following TLR and TNF-R1 stimulation of macrophages was the phosphorylation and activation of MKK3 and MKK6. Consequently, TPL-2 catalytic activity was required for the optimal activation of p38α, as well as ERK1/2. Phosphorylation of both MKK3 and MKK6 by TPL-2 was dependent on IKK2 catalytic activity and IKK2 phosphorylation of NF-κB1 p105, similar to TPL-2 phosphorylation of MKK1/2 [11,19]. Our findings therefore support a revised model of the signalling pathways controlling MAPK activation in innate immune responses (Figure 8), where stimulation of both p38α and ERK1/2 MAPKs is directly linked to the activation of NF-κB transcription factors via TPL-2. TPL-2 phosphorylation of MKK3/6 was also needed for optimal p38α activation following stimulation of macrophages with inactivated *M. tuberculosis* and *L. monocytogenes*, and probably contributes to the requirement for TPL-2 to mount effective innate immune responses to these two pathogens [17].

Although we clearly observed an essential role of TPL-2 kinase activity in the activation of MKK3/6 following stimulation of TLRs 3, 4, 9 and TNF-R1, there were quantitative differences in the effect of *Map3k8*^D270A^ mutation on downstream p38α activation according to the stimuli used. In fibroblasts, TNF stimulates MKK4 very weakly compared with UV [43]. Consequently, MKK4 functions redundantly with MKK3/6 for p38α activation following UV stimulation, but not TNF stimulation. Consistent with these results, we found that LPS induced a much higher degree of MKK4 activation loop phosphorylation than TNF in macrophages (data not shown). Consequently, MKK4 did not contribute substantially to TNF activation of p38α, and this was predominantly mediated by MKK3/6. In contrast, p38α was activated by MKK4 and MKK3/6 following LPS stimulation. Activation of MKK4 following LPS and TNF stimulation of macrophages was independent of TPL-2 catalytic activity. Quantitative differences in the degree of activation of MKK4 by LPS and TNF, therefore, provide a simple explanation for the distinct fractional reductions in p38α activation caused by *Map3k8*^D270A^ mutation in macrophages.

Comparison of the effects of *Map3k8*^D270A^ mutation with those of PD0325901 MKK1/2 inhibitor treatment revealed that TPL-2 largely regulated protein phosphorylation via activation of ERK1/2 in LPS-stimulated macrophages. Consistent with this conclusion, the TPL-2 phospho-motif deduced from phosphosites strongly dependent on TPL-2 catalytic activity indicated a strong preference for proline after the phosphorylated Ser or Thr residue [34]. However, there was also similarity at the −2 to −4 positions and +3 to +6 positions to the optimal phosphorylation motif for RSK [35], suggesting that a significant output of TPL-2 signalling was mediated by ERK1/2 activation of RSK. TPL-2 also regulated the phosphorylation of a few proteins independently of ERK1/2 activation, which included MKK3/6. We have previously shown that TPL-2 promotes the production of soluble TNF in LPS-stimulated macrophages independently of ERK1/2 activation [19]. However, since TPL-2 induces TNF production independently of IKK-induced p105 phosphorylation, this does not involve TPL-2 phosphorylation of MKK3/6, which is blocked by *Nfkb1*^SSAA/SSAA^ mutation. It is possible that other non-ERK1/2 phosphoproteins regulated by TPL-2 are involved in TPL-2 regulation of TNF production by macrophages and are potentially direct targets of TPL-2 catalytic activity.

TNF is a critical cytokine in inflammatory responses and innate immunity, regulating the production of cytokines, chemokines and prostaglandins. Moreover, dysregulated TNF is implicated in the pathogenesis of many autoimmune diseases, including rheumatoid arthritis, inflammatory bowel disease and psoriasis [51]. Biological agents that block TNF activity have been successfully used to treat these diseases. However, only a fraction of patients respond to TNF antibody therapy and there is still a need for more effective, less expensive, orally administered small molecule inhibitors to block TNF production and/or TNF biological activity. It is well established that TPL-2 regulates the production of TNF by TLR4-stimulated macrophages [14]. TPL-2 is also required for the response to TNF stimulation, regulating the activation of p38α, as shown in the present study, in addition to ERK1/2 [44]. Consequently, TPL-2 is an attractive drug target for TNF-dependent inflammatory diseases [16]. TPL-2 inhibition would be expected to have fewer side effects than pharmacological inhibition of MKK1/2, which would block ERK1/2 activation by TPL-2-independent receptors that activate MKK1/2 via Raf isoforms, such as growth factor, chemokine and antigen receptors. TPL-2 inhibitors may also be more effective than those directly targeting p38α, which have pro-inflammatory toxicities [52] due to blockade of negative feedback loops that suppress the activation of upstream regulatory kinases [53,54]. There is no evidence for similar negative feedback loops controlling TPL-2 signalling.

Understanding about the role of TPL-2 in regulating innate immune responses is largely limited to its effects on the expression of a few cytokines and chemokines in myeloid cells following TLR stimulation [6]. Functional classification of phosphosites reduced by *Map3k8*^D270A^ mutation in LPS-stimulated macrophages indicated that TPL-2 potentially controlled the function of proteins involved in many additional aspects of macrophage physiology. TPL-2-induced Rab11FIP5 phosphorylation, which is expected to promote activation of Rab11 via ERK1/2 [55], may control cargo transport via recycling endosomes to cell–cell junctions and phagosomes [56]. Inhibitory effects of *Map3k8*^D270A^ mutation on the phosphorylation of Fam21 [57], Dab2 [58] and Rab3/Rab3gap1 [59] also suggest that TPL-2 regulates vesicle trafficking. TPL-2 regulation of Ulk1 [60] and Wdfy3 (autophagy-linked FYVE protein) [61] phosphorylation raises the possibility that TPL-2 modulates autophagy. Given the important role of TPL-2 in innate immune responses [17] and the potential for TPL-2 as an anti-inflammatory drug target [16], it will be important in future studies to investigate the function of these and other TPL-2-regulated phosphorylations in macrophage biology and inflammatory responses.

In conclusion, our study has identified MKK3 and MKK6 as novel downstream targets of the IKK2/NF-κB1 p105/TPL-2 signalling pathway in TLR- and TNF-R1-stimulated macrophages. TPL-2 therefore mediates its essential role in innate immune responses via the stimulation of both p38α and ERK1/2 MAPKs, directly linking the activation of these MAPKs to the simultaneous activation of NF-κB transcription factors.

## Abbreviations

BMDM, bone marrow-derived macrophages; CpG, 5′-C-phosphate-G-3′; CREB, cyclic AMP response element-binding protein; CXCL, C-X-C motif ligand; ERK, extracellular signal-regulated kinase; FBS, foetal bovine serum; IFN, type I interferon; IKK, IκB kinase; IL-1R, interleukin-1 receptor; JNK, Jun amino terminal kinase; LPS, lipopolysaccharide; MAP, mitogen-activated protein; MAPK, MAP kinase; MAPKAP, MAPK-activated protein; MKK, MAPK kinase; MK2, MAPKAP kinase 2; MNK, MAPK-interacting kinase; MOI, multiplicity of infection; MSK, mitogen- and stress-activated protein kinase; NF-κB, nuclear factor κ-light-chain-enhancer of activated B cells; poly(I:C), polyinosinic–polycytidylic acid; RSK, p90 ribosomal S6 kinase; SEM, standard errors of the mean; SILAC, stable isotope amino acid cell culture; TAK1, transforming growth factor-β-activated kinase 1; TLRs, Toll-like receptors; TNF, tumour necrosis factor; TNF-R1, TNF receptor 1; TPL-2, tumour progression locus 2; UV, ultraviolet; WT, wild type; YB-1, Y-box 1.

## Author Contribution

M.J.P. co-designed and performed the majority of the immunoblotting experiments showing TPL-2 regulation of MAPK pathways, analyzed the phosphoproteome datasets and co-wrote the manuscript. O.M. prepared the macrophage lysates for mass spectrometric analysis, performed the initial analyses of phosphoproteome datasets and demonstrated TPL-2 regulation of MKK3/6 activation by immunoblotting. H.R.F. and A.P.S. co-designed the mass spectrometry experiments and carried out phosphoproteome analyses. C.-S.C. purified and performed MKK kinase assays with recombinant TPL-2^30-404^. H.-T.Y. and H.B.-A. carried out the preliminary experiments showing TPL-2 regulation of MKK3/6 phosphorylation. S.B. generated the heatmaps and TPL-2 phospho-motifs from the phosphoproteome datasets. S.C.L. initiated the study, co-designed the experiments and co-wrote the manuscript.

## Funding

This research was supported by core funding from The Francis Crick Institute (UK).
